# The impact of thoracic duct resection on the long-term body composition of patients who underwent esophagectomy for esophageal cancer and survived without recurrence

**DOI:** 10.1093/dote/doad002

**Published:** 2023-07-18

**Authors:** Erica Nishimura, Satoru Matsuda, Hirofumi Kawakubo, Jun Okui, Ryo Takemura, Masashi Takeuchi, Kazumasa Fukuda, Rieko Nakamura, Hiroya Takeuchi, Yuko Kitagawa

**Affiliations:** Department of Surgery, Keio University School of Medicine, Tokyo, Japan; Department of Surgery, Keio University School of Medicine, Tokyo, Japan; Department of Surgery, Keio University School of Medicine, Tokyo, Japan; Department of Surgery, Keio University School of Medicine, Tokyo, Japan; Department of Preventive Medicine and Public Health, Keio University School of Medicine, Tokyo, Japan; Biostatistics Unit, Clinical and Translational Research Center, Keio University School of Medicine, Tokyo, Japan; Department of Surgery, Keio University School of Medicine, Tokyo, Japan; Department of Surgery, Keio University School of Medicine, Tokyo, Japan; Department of Surgery, Keio University School of Medicine, Tokyo, Japan; Department of Surgery, Hamamatsu University School of Medicine, Shizuoka, Japan; Department of Surgery, Keio University School of Medicine, Tokyo, Japan

**Keywords:** esophageal cancer, esophagectomy, nutrition, quality of life

## Abstract

Background: We have reported the possible benefits of radical esophagectomy with thoracic duct (TD) resection in elective esophageal cancer surgery. However, the effect of TD resection on the long-term nutrition status remains unclear. Methods: Patients who underwent esophagectomy at Keio University between January 2006 and December 2018 were included, and those who had no recurrence for more than three years were evaluated. Changes in each body composition (muscle mass and body fat) were comparatively assessed between those who underwent TD resection or not, before and at, one, three and five years after surgery. Computed tomography images were analyzed on postoperative year 1, 3 and 5. Results: This study included 217 patients categorized in the TD-resected (TD-R) (156 patients) and TD-preserved (TD-P) (61 patients) groups. The loss of muscle mass was comparable between the groups. On the other hand, the loss of adipose tissues was significantly greater in the TD-R group than in the TD-P group at one and three years after surgery, while there was no statistical difference five years after surgery. Additionally, among patients with cT1N0M0 disease in whom survival advantage of TD resection has been reported previously, the loss of muscle mass did not differ between each group. Conclusions: The change of muscle mass between the two groups was comparable. Although body fat mass was reduced by TD resection, it eventually recovered in the long term. In patients with esophageal cancer, TD resection may be acceptable without significant impact on body composition in the long term.

## INTRODUCTION

Esophagectomy is considered to be one of the standard treatments for esophageal cancer.^1^ The Japanese guideline recommended three-field lymphadenectomy for thoracic esophageal cancer, which has a high frequency of extensive lymph node (LN) metastasis.^2^ We previously reported the improved survival of patients with cStage I esophageal cancer after esophagectomy with extensive LN dissection along with TD dissection.^3^ In addition, it has been suggested that routine nodal dissection with TD resection should be performed, because the incidence of LN metastasis in thoracic duct nodes was related with the tumor depth.^4^ Recently, another group reported that dissection of the TD LNs improved long-term survival and had similar effectiveness with dissection of other regional LNs.^5^ Moreover, it has been reported that TD resection may improve prognosis by reducing hematogenous recurrence.^6^ On the contrary, a recent study claimed that although TD resection was accompanied with a relatively high number of retrieved mediastinal LNs and relatively less LN recurrence, it did not improve the survival of patients with esophageal cancer.^7^ Overall, the survival benefit of resection of the thoracic duct (TD) and its surrounding adipose tissue remains uncertain.

The impact of TD resection on nutritional status has been another concern.^8^ It has been reported that TD resection decreased body fat mass, but the loss of muscle mass was comparable to the procedure.^9^ Although this study included patients with recurrence, to exclude the confounding effect of deteriorated nutrition status by cancer cachexia,^10^ it is preferred that the study is conducted on patients who are cancer free. Furthermore, in order to evaluate the impact on body composition, longer follow-up would be desirable. In this study, we aimed to evaluate the long-term impact of TD resection on body composition by analyzing patients who had no recurrence in the first three years after esophagectomy for esophageal cancer.

## METHODS

### Patients

This was a retrospective analysis from a single institution. We identified patients who underwent radical resection for thoracic esophageal cancer at the Keio University between January 2006 and December 2018 and survived without any recurrence for three years after surgery, since esophageal cancer commonly recurs within three years after surgery.^11–13^ Patients who were lost to follow-up, did not undergo gastric tube reconstruction, received preoperative chemoradiotherapy, or stayed at the hospital for longer than six months were excluded. In Japan, the standard treatment of patients diagnosed with a surgically resectable stage of cancer is upfront surgery or neoadjuvant chemotherapy and surgery^2^; those who undergo chemoradiation therapy are usually thought to have initially unresectable disease. In this study, our inclusion criteria were those believed to have surgical resectability. Therefore, to exclude confounding factors, we excluded patients who received chemoradiation therapy. Before treatment, patients were evaluated by esophagoscopy and computed tomography (CT), with the addition of positron emission tomography from the year 2013. Clinical staging and pathologic examination of the tumors were performed according to the TNM classification, 8th edition.^14^ Postoperative complications were evaluated using the Clavien–Dindo classification.^15^

### Perioperative therapy, surgical procedure, and follow-up

A multidisciplinary conference was held to verify the validity of the surgery indication for each patient. Based on the Japan Clinical Oncology Group (JCOG) 9907 study, neoadjuvant chemotherapy, using cisplatin and 5-fluorouracil, was the standard treatment since 2007.^11^Until then, those who were thought to be resectable underwent upfront surgery. Esophagectomy was performed following a right-sided thoracic approach with gastric tube reconstruction. Most patients underwent reconstruction through the posterior mediastinal route. Between 2006 and 2008, esophagectomy without TD resection was adopted as a standard surgical procedure at our institution. From 2009, esophagectomy with TD resection was carried out to achieve a more radical dissection.^3^ TD resection was not performed on patients who had liver cirrhosis, kidney disease, or heart disease, because it was reported to have a possible influence on postoperative fluid retention and liver function.^16^ LNs in the following areas were routinely dissected: mediastinal, bilateral recurrent laryngeal nerve, abdominal, pericardial, lesser curvature, and left gastric artery. Feeding jejunostomy during surgery was routinely performed, and tube feeding via a jejunostomy was started immediately after esophagectomy. In the follow-up evaluations, esophagoscopy and CT were performed every six months for five years after surgery.

### CT analysis of body composition

Body composition was assessed by analyzing the CT images obtained with the SYNAPSE VINCENT Volume Analyzer (Fujifilm Medical Co., Tokyo, Japan). This image analyzer software enabled easy and accurate reconstruction of three-dimensional images of the visceral/subcutaneous fat and psoas major muscle (Supplementary Figure S1).

The abdominal CT images were acquired preoperatively and at one and three years after surgery. CT images at five years after surgery were also analyzed if possible. We assessed the psoas muscle, subcutaneous adipose tissue (SAT), and visceral adipose tissue (VAT) at the level of the third lumbar vertebra.^17^ The psoas muscle was identified and quantified by a Hounsfield of −29 to 150, whereas the SAT and VAT were identified and quantified by a Hounsfield unit threshold of −190 to −30 and − 150 to −50, respectively.^18^ Psoas muscle was evaluated by the psoas muscle index (PMI), which has been used as a parameter for evaluating the skeletal muscle mass of the whole body.^19^ The PMI was calculated as follows:

L3 PMI (cm^2^/m^2^) = L3 psoas muscle cross-sectional area (cm^2^)/height^2^ (m^2^).

### Evaluation of the loss of body composition

The percentage of SAT, VAT, and PMI were calculated as follows:

SAT = (SAT at a certain time point/preoperative SAT) × 100

VAT = (VAT at a certain time point/preoperative VAT) × 100

PMI = (PMI at a certain time point/preoperative PMI) × 100

### Analysis of the effects of TD resection on the changes in body composition

According to whether or not the surgery proceeded to TD resection, the patients were divided into the following two groups: TD-resected (TD-R) and TD-preserved (TD-P) groups.

**Fig. 1:**
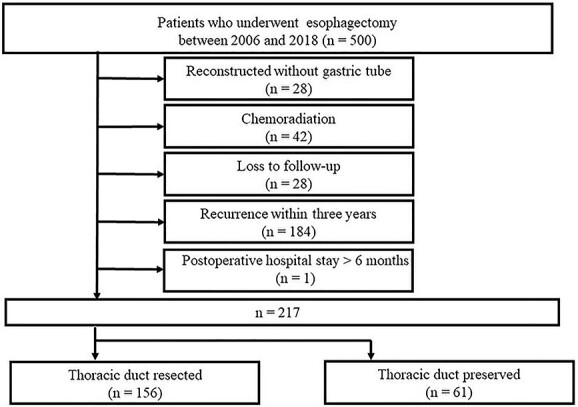
CONSORT diagram of this study. Patients who were survived without any recurrence for 3 years after esophagectomy were included (*n* = 217).

### Statistical analysis

IBM SPSS for Windows (version 27, Armonk, NY, USA) was used for data analysis. Statistical tests were used when required to determine correlations between variables. The chi-square and Fisher’s exact test was used to compare categorical variables. For the comparison of continuous variables, the Student’s *t-*test was used for variables with normal distribution, whereas the Mann–Whitney U test was used for variables without normal distribution. Statistical significance was set at *P* < 0.05. Multiple logistic regression analysis was performed using binary logistic regression to determine the risk factors for the loss of each body composition (i.e. greater than each median at each time point). We produced survival curves of noncancer-specific survival using the Kaplan–Meier survival method. We defined death associated with comorbidity when the patient died because of a nontumoral disease and in the absence of evidence of the tumor. Cancer related death was treated as censored observation. The data that support the findings of this study are available from the corresponding author upon reasonable request.

## RESULTS

Of the 500 patients who underwent esophagectomy for esophageal cancer during the study period, 28 who did not undergo gastric tube reconstruction, 42 who received preoperative CRT, 28 who were lost to follow-up, 184 who had cancer recurrence within three years, and 1 who stayed at the hospital for longer than six months were excluded. Finally, 217 patients were included in this study; 156 were categorized into the TD-R group, whereas the remaining 61 were categorized into the TD-P group. A CONSORT flow diagram is depicted in Fig. 1. The body composition of these patients was analyzed after one and three years after surgery. Since the study period was from January 2006 to December 2018, some did not reach the 5-year follow-up period in this study. Among 171 patients who underwent surgery before 2017, for whom who should meet their 5-year follow-up, 15 patients were lost to follow-up 3 years after surgery, 2 were found with recurrence, and 2 were died from non-cancer death. Finally, we analyzed the body composition of 152 patients at this time point (TD-R 106 patients, TD-P 46 patients). Two patients who were categorized in the TD-R group and were found to have recurrence on postoperative year (POY) 3.5 were also included in this study.

The clinical characteristics of the patients in each group are shown in Table 1. No significant differences were noted between the groups with regard to age and sex. The surgical outcomes of the study are shown in Table 2. Although the operation time was significantly longer in the TD-R group than in the TD-P group, there was no significant difference in intraoperative blood loss between the groups. More patients underwent thoracoscopic surgery in the TD-R group than in the TD-P group (96 vs. 67%; *P* < 0.001). The incidence of postoperative complications, including anastomotic leakage, recurrent laryngeal nerve palsy, and pneumonia, as well as length of postoperative hospital stay, was not significantly different between the groups.

**Table 1:** Patient characteristics

**Variable**		**TD resected (*n* = 156)**	**TD preserved (*n* = 61)**	***P* value**
Age^*^		64 (58–70)	62 (56–67.5)	0.243
Sex (male/female)		128/28 (82%/18%)	50/11 (82%/18%)	0.988
Location				0.329
	Ce	1 (0.6%)	0 (0%)	
	Ut	17 (11%)	9 (15%)	
	Mt	80 (51%)	27 (44%)	
	Lt	46 (29%)	17 (28%)	
	Ae	12 (7.7%)	8 (13%)	
Neoadujuvant chemotherapy		80 (51%)	19 (31%)	0.007
Pathological depth of invasion				0.271
	p T0	12 (7.7%)	4 (6.6%)	
	p T1	97 (62%)	34 (56%)	
	p T2	17 (11%)	5 (8.2%)	
	p T3	30 (19%)	17 (28%)	
	p T4	0 (0.0%)	1 (1.6%)	
Pathological lymph node metastasis				0.002
	p N0	92 (59%)	45 (74%)	
	p N1	44 (28%)	12 (20%)	
	p N2	17 (11%)	4 (6.6%)	
	p N3	3 (1.9%)	0 (0.0%)	
Pathological distant metastasis				0.569
	p M0	149 (96%)	58 (95%)	
	p M1	7 (4.5%)	3 (4.9%)	
Pathological stage				0.262
	p Stage 0/I	71 (46%)	29 (48%)	
	p Stage II	42 (27%)	22 (36%)	
	p Stage III	34 (22%)	6 (9.8%)	
	p Stage IV	9 (5.8%)	4 (6.6%)	

^*^Data are presented as median (IQR).

TD, thoracic duct

**Table 2:** Surgical procedure and postoperative course

**Variable**		**TD resected (*n* = 156)**	**TD preserved(*n* = 61)**	***P* value**
Operation time (mins)^*^		508 (471–565)	483 (415–528)	0.002
Intraoperative blood loss (mL)^*^		150 (61.3–230)	180 (80–350)	0.063
Surgical procedure				<0.001
	Open	6 (3.8%)	20 (33%)	
	Thoracoscopic surgery	149 (96%)	41 (67%)	
	Robot	1 (0.6%)	0 (0%)	
Postoperative complications				
	Anastomotic leakage (≥CD III)	21 (13%)	8 (13%)	0.946
	RLN palsy (≥CD III)	7 (4.5%)	0 (0%)	0.093
	Pneumonia (≥CD II)	26 (17%)	6 (9.8%)	0.202
Postoperative hospital stay (day)^*^		25 (21–34.8)	27 (21–36)	0.282

^*^Data are presented as median (IQR).

TD, thoracic duct; CD, Clavien–Dindo; RLN, recurrent laryngeal nerve

**Fig. 2:**
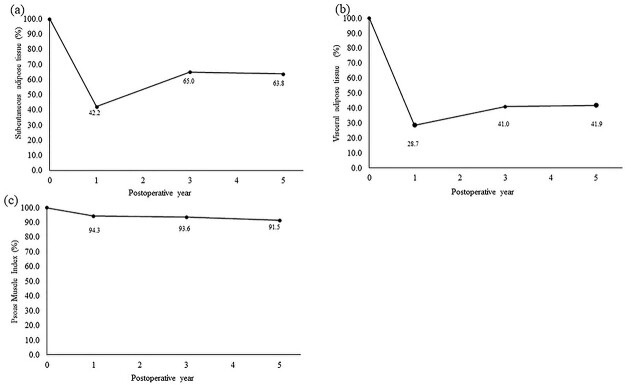
Longitudinal changes in body composition of all patients: a: subcutaneous adipose tissue; b: visceral adipose tissue and c: psoas muscle index. Individual plot indicates median value.

The longitudinal difference in each body composition is presented in Fig. 2. The loss of PMI was smaller than the loss of fat, and it was maintained throughout the observation period. On the other hand, the losses of SAT and VAT were substantial at one year after surgery. Notably, the loss of VAT was greater than that of SAT throughout the entire observation period. The losses of SAT and VAT improved within three years and plateaued thereafter.

**Fig. 3:**
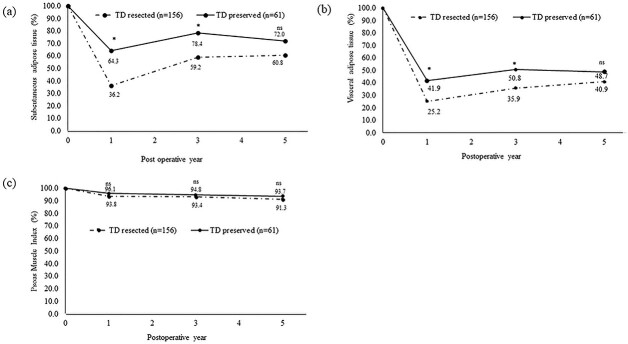
Comparison of the longitudinal changes in body composition between the TD-resected and the TD-preserved groups: a: subcutaneous adipose tissue; b: visceral adipose tissue and c: psoas muscle index. ^*^P < 0.05 individual plot indicates median value. TD, thoracic duct; ns, not significant.

Figure 3 compares the decrease in each body composition between the TD-R and TD-P groups. First, PMI was maintained until five years after surgery and did not differ between the two groups. Next, although both SAT and VAT showed considerable decreases at one year after surgery, both were significantly decreased in the TD-R group than in the TD-P group (SAT: TD-R 36.2% vs. TD-P 64.3%, *P* < 0.001/VAT: TD-R 25.2% vs. TD-P 41.9%, *P* < 0.001). Although the SAT and VAT increased at three years after surgery, compared with the TD-P group, the TD-R group showed significantly greater loss of SAT (59.2 vs. 78.4%; *P* = 0.003) and VAT (35.9 vs. 50.8%; *P* = 0.01). However, at five years after surgery, the TD-R and -P groups had similar loss of SAT (60.8 vs. 72.0%, respectively; *P* = 0.07) and VAT (40.9 vs. 48.7%, respectively; *P* = 0.456). The change of each body composition (median and IQR) at each time point is presented in supplementary table S1. To determine the factors responsible for loss of SAT and VAT greater than median at each time point, univariate and multivariate analyses were performed. The results of the loss of body fat at one and three years after surgery are shown in Tables 3 and 4, respectively. The loss of SAT was affected by TD resection, whereas the loss of VAT was affected by age ≥ 75 years and TD resection. Additionally, we compared the change of each body composition among patients with cT1N0M0 disease and had not undergone neoadjuvant chemotherapy, whom we have previously reported to have had an oncological benefit from TD resection (total of 108 patients; TD-R 73 patients, TD-P 35 patients).^3^ As for PMI, although the PMI maintained in both groups throughout the study period, there was no statistical difference in any time point. Considering body fat, the TD-R group showed a significantly greater loss in both SAT and VAT, but only on POY 1, and the change was comparable beyond POY 3 (data not shown). Finally, we compared the non-cancer related survival was comparable between the TD-R and -P groups (5 year OS: TD-R 99.2% vs. TD-P 100%, *P* = 0.916, Supplementary Figure S2).

**Table 3:** Summary of logistic regression analysis of the predictors of change in body fat mass one year after surgery

**(a) Subcutaneous fat**						
**Variable**	**Univariate analysis**			**Multivariate analysis**		
	**OR**	***P* value**	**95% CI**	**OR**	***P* value**	**95% CI**
Age ≥ 75 years	1.55	0.36	0.61–3.95	1.82	0.23	0.68–4.85
Sex (male)	0.74	0.40	0.37–1.48	0.70	0.34	0.34–1.45
Neoadujuvant chemotherapy	0.76	0.31	0.44–1.29	0.64	0.13	0.36–1.14
Pathological lymph node metastasis, pN ≥1	1.07	0.82	0.61–1.85	1.01	0.97	0.56–1.83
Thoracoscopic surgery	1.72	0.21	0.74–4.00	1.1290	0.82	0.43–2.90
Thoracic duct resection	2.24	0.010	1.21–4.12	2.49	0.010	1.24–5.01
**(b) Visceral fat**						
Age ≥ 75 years	1.95	0.17	0.75–5.11	2.88	0.047	1.01–8.15
Sex (male)	1.79	0.11	0.88–3.64	1.77	0.14	0.83–3.77
Neoadujuvant chemotherapy	0.61	0.067	0.35–1.04	0.56	0.058	0.30–1.02
Pathological lymph node metastasis, pN ≥1	0.61	0.083	0.35–1.07	0.55	0.058	0.30–1.02
Thoracoscopic surgery	2.53	0.039	1.05–6.09	1.40	0.51	0.51–3.86
Thoracic duct resection	2.72	0.002	1.46–5.07	3.46	<0.001	1.67–7.20

**Table 4:** Summary of logistic regression analysis of the predictors of change in body fat mass three years after surgery

**(a) Subcutaneous fat**						
**Variable**	**Univariate analysis**			**Multivariate analysis**		
	**OR**	***P* value**	**95% CI**	**OR**	***P* value**	**95% CI**
Age ≥ 75 years	0.99	0.983	0.40–2.48	1.29	0.61	0.49–3.41
Sex (male)	1.08	0.835	0.54–2.15	1.03	0.935	0.50–2.13
Neoadujuvant chemotherapy	0.76	0.31	0.44–1.29	0.70	0.230	0.39–1.26
Pathological lymph node metastasis, pN ≥1	0.63	0.145	0.38–1.15	0.60	0.098	0.33–1.10
Thoracoscopic surgery	2.08	0.095	0.88–4.89	1.29	0.85	0.50–3.41
Thoracic duct resection	2.72	0.002	1.46–5.07	3.18	0.001	1.56–6.48
**(b) Visceral fat**						
Age ≥ 75 years	3.29	0.026	1.15–9.39	4.40	0.009	1.44–13.5
Sex (male)	1.57	0.21	0.78–3.18	1.53	0.26	0.74–3.18
Neoadujuvant chemotherapy	0.82	0.46	0.48–1.39	0.76	0.36	0.42–1.37
Pathological lymph node metastasis, pN ≥1	0.91	0.74	0.52–1.58	0.80	0.47	0.44–1.47
Thoracoscopic surgery	2.08	0.095	0.88–4.89	1.50	0.26	0.74–3.18
Thoracic duct resection	2.24	0.010	1.21–4.12	2.48	0.012	1.22–5.03

## DISCUSSION

In this study, the PMI was maintained for five years, and more importantly, it did not differ significantly between the groups. On the other hand, for three years after the surgery, decrease in body fat mass was relatively high in the TD-R group and TD resection was a risk factor for loss of body fat. However, fat loss in the TD-R group improved and became comparable with that in the TD-P group within five years after surgery. In addition, the incidence of postoperative complications secondary to TD resection did not increase. Based on these results, TD resection in patients with esophageal cancer may be acceptable, with no significant long-term impact on body composition.

So far, only few studies have analyzed change in body composition as a tool to define the nutritional status after TD resection for radical esophagectomy. Fujisawa et al compared the changes in the body composition before and one year after esophagectomy, with or without TD resection^9^; interestingly, the trend of decrease in body composition in their study was similar with the trend in our study. In the TD-R group, body fat mass decreased in the first three years after surgery but recovered after five years. To the best of our knowledge, this was the first study to report that although body fat decreased after TD resection, it can recover in the long term. After a fatty meal, dietary lipids enter the venous system in the small intestine through the TD^20^; therefore, the association of TD resection with loss of fat mass in the short term after surgery was reasonable. Eventually, lymphatic collateral circulation develops after TD resection,^21^ and this can explain the improvement seen within five years after surgery.

Moreover, it is important to note that TD resection did not have influence on the loss of PMI throughout the study period. Although there are few reports that claim that body fat is responsible for a deteriorated quality of life, muscle wasting after surgery has been multivalley reported to be associated with poor quality of life ^22^^,^^23^ Based on our results, TD resection did not affect loss of muscle mass and noncancer-related survival. Considering these facts, curative-intent TD resection may be acceptable in patients with esophageal cancer. We have reported that TD resection may improve survival of patients with cT1N0M0 disease and without neoadjuvant chemotherapy.^3^ Interestingly, the change of muscle mass was not affected by TD resection in both the short and the long terms. As for body fat, the loss was only comparable with POY1. There is a possibility that the change of body composition in this population reflects the influence of TD resection strongly, because their influence of cancer cachexia must be lower because of the early stage and that they did not receive neoadjuvant therapy. The fact that body composition did not differ greatly in this population strengthens our inference that TD resection may be acceptable for survival and long-term QOL.

In comparing SAT and VAT, we found that the loss of VAT was greater than that of SAT. Although we did not fully investigate the correlation between VAT and SAT, previous researchers reported that the change in VAT was greater than that in SAT after gastrectomy, which may indicate a greater influence on VAT after gastrointestinal surgery than SAT.^18^ Finally, we also evaluated the change in body composition 1 year after surgery for those who had recurrence within 3 years after surgery (TD-R with recurrence: 58 patients, TD-P with recurrence: 45 patients). Interestingly, there were no significant differences between the TD-R and TD-P groups (data not shown). Since patients who experience recurrence are at risk for malnutrition due to the cancer itself, TD resection for patients who experience recurrence may have less of an effect on body composition.

Because TD was preserved in patients with comorbidities before surgery since 2009, it might be expected that the TD-P group would show a greater incidence of noncancer death. However, as shown in Supplementary Figure 2, we have analyzed the noncancer death rate and found it to be comparable between the two groups (5 year overall survival: TD-R 99.2% vs. TD-P 100%, *P* = 0.916). We speculated that despite the existence of preoperative comorbidity, all patients in this study met the criteria to undergo transthoracic esophagectomy, and their inclusion did not largely influence the incidence of noncancer death.

In this study, body composition was evaluated by analyzing CT images, not by bioelectrical impedance. This enables us to assess muscle mass and body fat more objectively. The change of skeletal mass is included in the definition of sarcopenia.^24^ Sarcopenia is not only reported as an unfavorable prognostic factor,^25^^,^^26^ but is also associated with reduction in both physical and mental health-related quality of life.^27^ Because a previous report claimed that loss of body fat predicts poor survival,^17^ we believe that clarifying the change of body composition has high clinical validity. On the contrary, it is unclear whether patients included in this study with greater loss of each body composition experience an increasingly deteriorated quality of life. Quality of life should be assessed with various angles, and further research including functional assessment and patient-reported outcome should be focused upon in the future.

The present study had several limitations. First, given that this was a retrospective study conducted at a single institution, selective bias for the indications of surgery could not be excluded. In fact, TD resection was not performed on patients with liver cirrhosis, kidney disease, or heart disease. However, these patients were also at risk for decrease in body composition, and the fact that loss of fat mass was relatively high after TD resection strengthened our findings that TD resection had an influence on the decrease in adipose tissue. Second, more patients received preoperative therapy in the TD-R group than in the TD-P group. Because chemotherapy promotes changes in body composition,^28^ the results obtained may have been biased. Nevertheless, we compared the change in each body composition between groups that included patients who did not receive preoperative therapy and found the same trend, except for the change in VAT at three years after surgery (data not shown). In addition, our treatment strategy changed to resect TD from 2009, and TD was preserved in most patients who underwent esophagectomy before 2008. Although the perioperative management, including enteral nutrition, was consistent throughout the entire study period, this may cause a selection bias.

In conclusion, TD resection was not related with the short- and long-term loss of muscle mass after esophagectomy. Although body fat mass decreased in the short term after TD resection, it improved in the long term, and TD resection did not show any significant effect at POY 5. In patients with esophageal cancer, TD resection may be acceptable without significant long-term impact on body composition.

## ABBREVIATIONS

TD thoracic duct, TD-P thoracic duct-preserved group, TD-R thoracic duct-resected group, SAT subcutaneous adipose tissue, VAT visceral adipose tissue, PMI Psoas muscle index

## Supplementary Material

Supplement_Figure_1_doad002

Supplement_Figure_2_doad002
