# Supplementary figures and images for: The impact of thoracic duct resection on the long-term body composition of patients who underwent esophagectomy for esophageal cancer and survived without recurrence
Source: Dis Esophagus. 2023 Jul 18;36(9):doad002. doi: 10.1093/dote/doad002 (PMC10473448; doi:10.1093/dote/doad002)

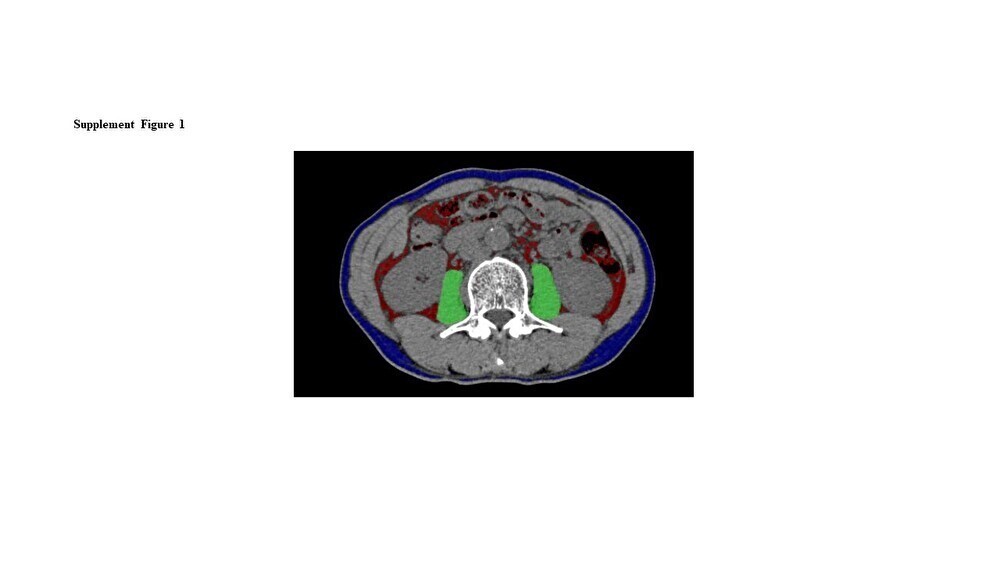

Supplement: Supplement_Figure_1_doad002 [file supplement_figure_1_doad002.jpeg]

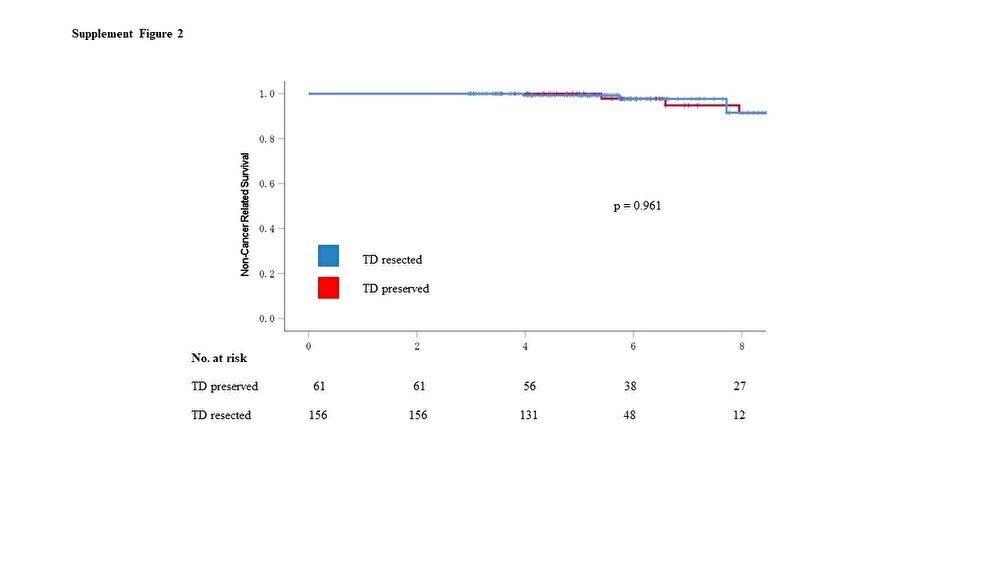

Supplement: Supplement_Figure_2_doad002 [file supplement_figure_2_doad002.jpeg]
